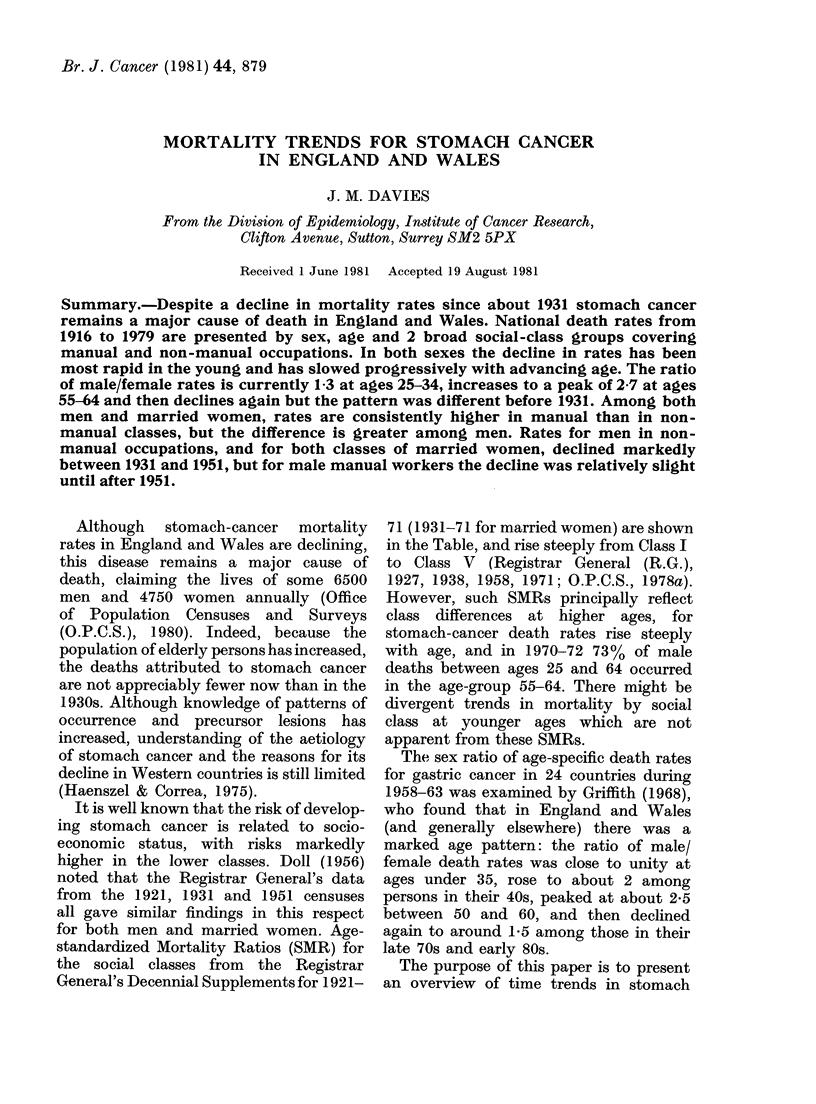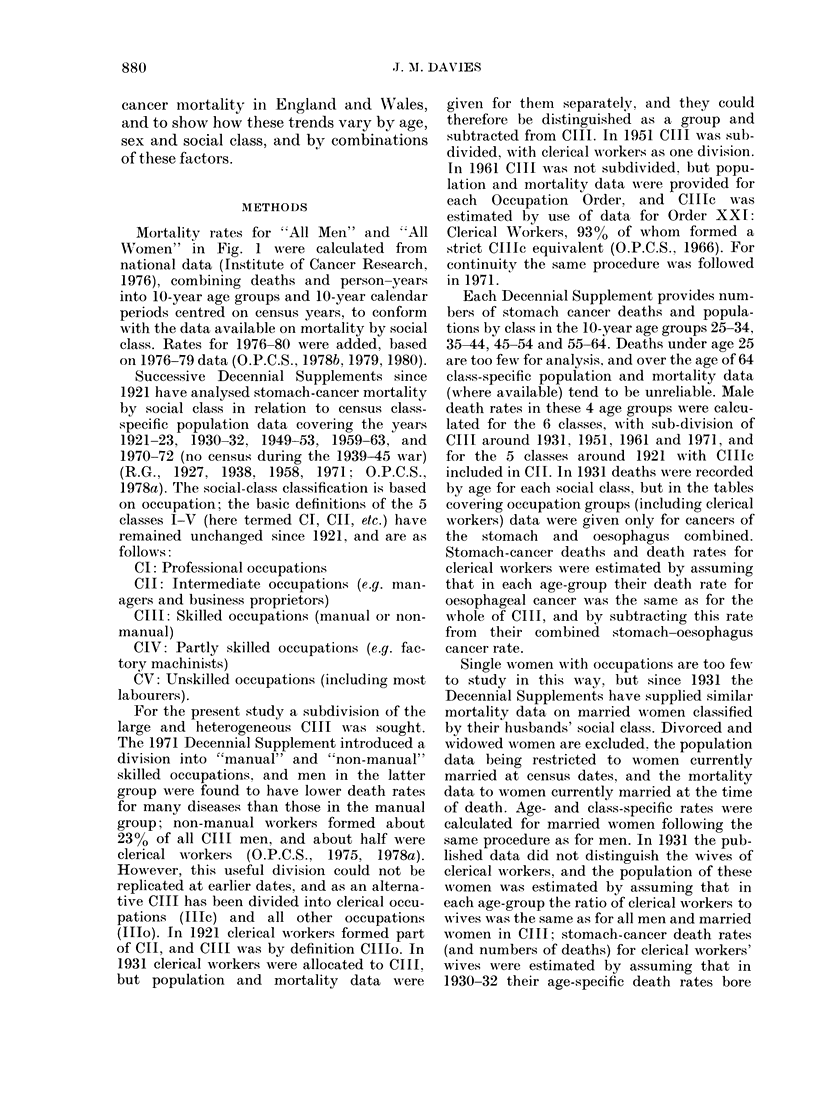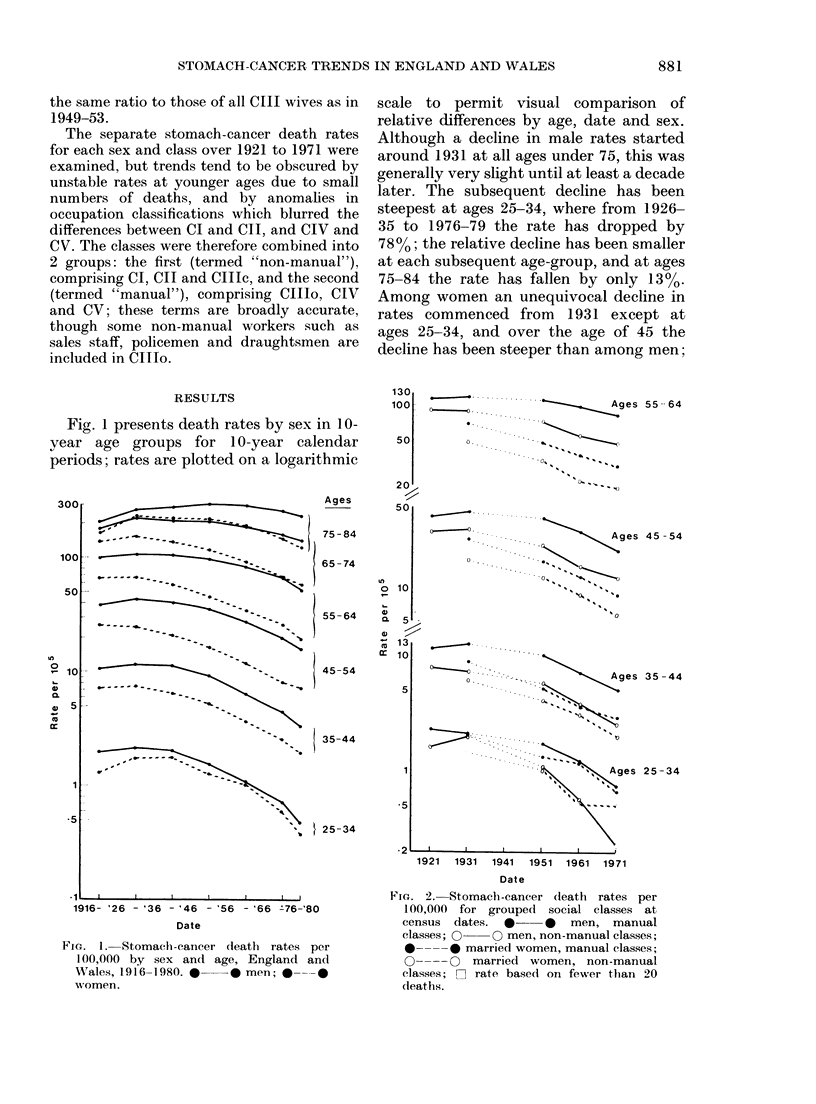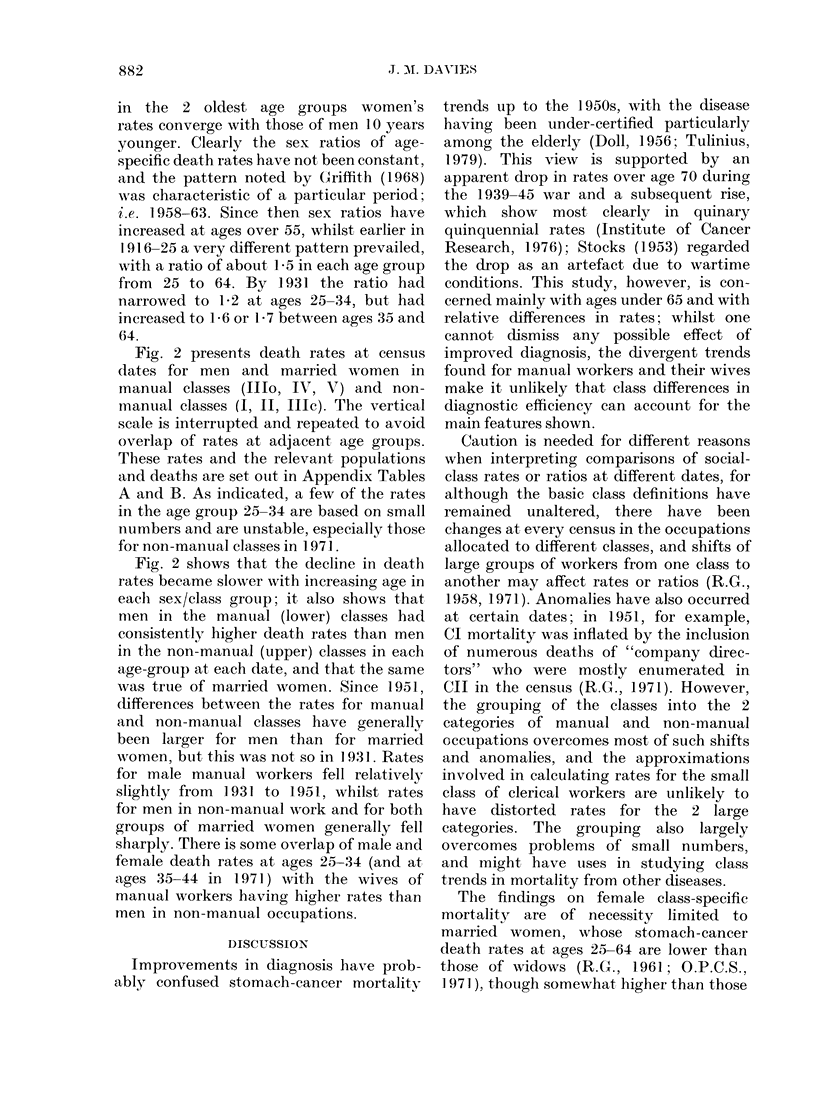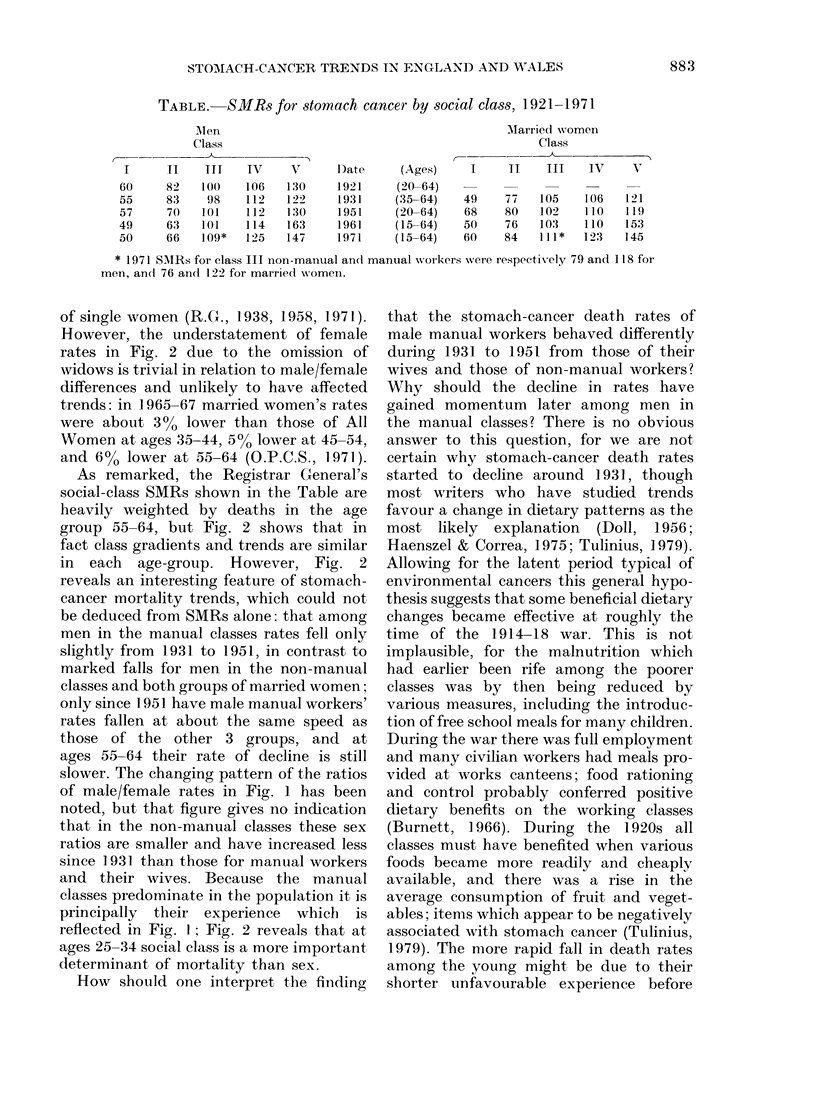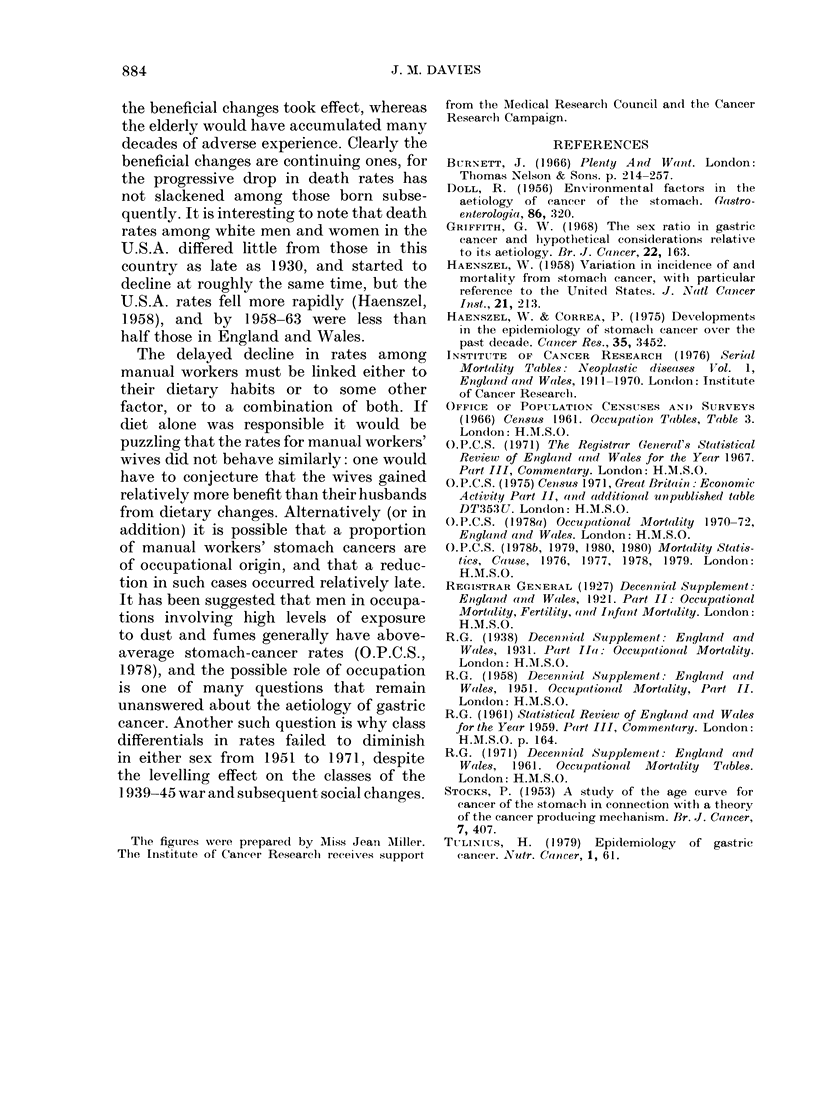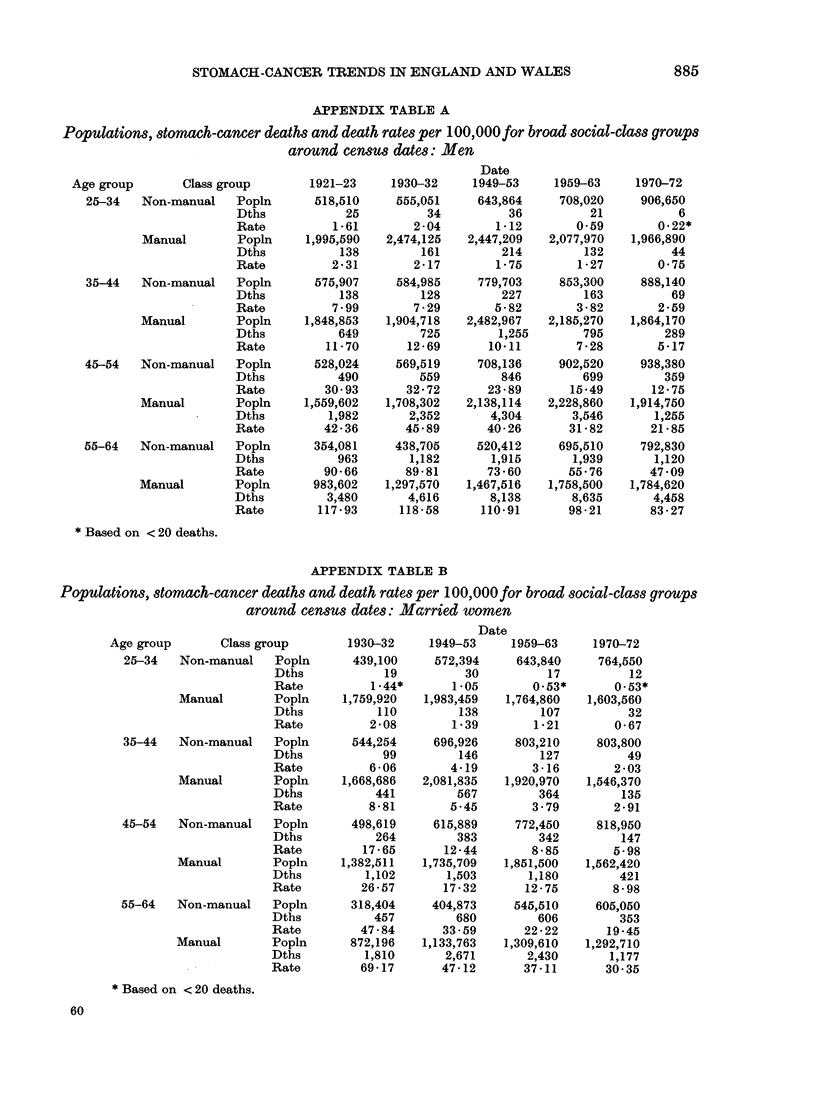# Mortality trends for stomach cancer in England and Wales.

**DOI:** 10.1038/bjc.1981.287

**Published:** 1981-12

**Authors:** J. M. Davies

## Abstract

Despite a decline in mortality rates since about 1931 stomach cancer remains a major cause of death in England and Wales. National death rates from 1916 to 1979 are presented by sex, age and 2 broad social-class groups covering manual and non-manual occupations. In both sexes the decline in rates has been most rapid in the young and has slowed progressively with advancing age. The ratio of male/female rates is currently 1.3 at ages 25-34, increases to a peak of 2.7 at ages 55-64 and then declines again but the pattern was different before 1931. Among both men and married women, rates are consistently higher in manual than in non-manual classes, but the difference is greater among men. Rates for men in non-manual occupations, and for both classes of married women, declined markedly between 1931 and 1951, but for male manual workers the decline was relatively slight until after 1951.


*Br. J. Cancer* (1981) **44**, 879

### MORTALITY TRENDS FOR STOMACH CANCER IN ENGLAND AND WALES

J. M. DAVIES

*From the Division of Epidemiology, Institute of Cancer Research, Clifton Avenue, Sutton, Surrey SM2 5PX*

Received 1 June 1981 Accepted 19 August 1981

**Summary.—Despite a decline in mortality rates since about 1931 stomach cancer remains a major cause of death in England and Wales. National death rates from 1916 to 1979 are presented by sex, age and 2 broad social-class groups covering manual and non-manual occupations. In both sexes the decline in rates has been most rapid in the young and has slowed progressively with advancing age. The ratio of male/female rates is currently 1·3 at ages 25–34, increases to a peak of 2·7 at ages 55–64 and then declines again but the pattern was different before 1931. Among both men and married women, rates are consistently higher in manual than in non-manual classes, but the difference is greater among men. Rates for men in non-manual occupations, and for both classes of married women, declined markedly between 1931 and 1951, but for male manual workers the decline was relatively slight until after 1951.**

Although stomach-cancer mortality rates in England and Wales are declining, this disease remains a major cause of death, claiming the lives of some 6500 men and 4750 women annually (Office of Population Censuses and Surveys (O.P.C.S.), 1980). Indeed, because the population of elderly persons has increased, the deaths attributed to stomach cancer are not appreciably fewer now than in the 1930s. Although knowledge of patterns of occurrence and precursor lesions has increased, understanding of the aetiology of stomach cancer and the reasons for its decline in Western countries is still limited (Haenszel & Correa, 1975).

It is well known that the risk of developing stomach cancer is related to socio-economic status, with risks markedly higher in the lower classes. Doll (1956) noted that the Registrar General's data from the 1921, 1931 and 1951 censuses all gave similar findings in this respect for both men and married women. Age-standardized Mortality Ratios (SMR) for the social classes from the Registrar General's Decennial Supplements for 1921–71 (1931–71 for married women) are shown in the Table, and rise steeply from Class I to Class V (Registrar General (R.G.), 1927, 1938, 1958, 1971; O.P.C.S., 1978*a*). However, such SMRs principally reflect class differences at higher ages, for stomach-cancer death rates rise steeply with age, and in 1970–72 73% of male deaths between ages 25 and 64 occurred in the age-group 55–64. There might be divergent trends in mortality by social class at younger ages which are not apparent from these SMRs.

The sex ratio of age-specific death rates for gastric cancer in 24 countries during 1958–63 was examined by Griffith (1968), who found that in England and Wales (and generally elsewhere) there was a marked age pattern: the ratio of male/female death rates was close to unity at ages under 35, rose to about 2 among persons in their 40s, peaked at about 2·5 between 50 and 60, and then declined again to around 1·5 among those in their late 70s and early 80s.

The purpose of this paper is to present an overview of time trends in stomach cancer mortality in England and Wales, and to show how these trends vary by age, sex and social class, and by combinations of these factors.

### METHODS

Mortality rates for "All Men" and "All Women" in Fig. 1 were calculated from national data (Institute of Cancer Research, 1976), combining deaths and person–years into 10-year age groups and 10-year calendar periods centred on census years, to conform with the data available on mortality by social class. Rates for 1976–80 were added, based on 1976–79 data (O.P.C.S., 1978*b*, 1979, 1980).

Successive Decennial Supplements since 1921 have analysed stomach-cancer mortality by social class in relation to census class-specific population data covering the years 1921–23, 1930–32, 1949–53, 1959–63, and 1970–72 (no census during the 1939–45 war) (R.G., 1927, 1938, 1958, 1971; O.P.C.S., 1978*a*). The social-class classification is based on occupation; the basic definitions of the 5 classes I–V (here termed CI, CII, *etc.*) have remained unchanged since 1921, and are as follows:

CI: Professional occupations

CII: Intermediate occupations (*e.g.* managers and business proprietors)

CIII: Skilled occupations (manual or non-manual)

CIV: Partly skilled occupations (*e.g.* factory machinists)

CV: Unskilled occupations (including most labourers).

For the present study a subdivision of the large and heterogeneous CIII was sought. The 1971 Decennial Supplement introduced a division into "manual" and "non-manual" skilled occupations, and men in the latter group were found to have lower death rates for many diseases than those in the manual group; non-manual workers formed about 23% of all CIII men, and about half were clerical workers (O.P.C.S., 1975, 1978*a*). However, this useful division could not be replicated at earlier dates, and as an alternative CIII has been divided into clerical occupations (IIIc) and all other occupations (IIIo). In 1921 clerical workers formed part of CII, and CIII was by definition CIIIo. In 1931 clerical workers were allocated to CIII, but population and mortality data were given for them separately, and they could therefore be distinguished as a group and subtracted from CIII. In 1951 CIII was subdivided, with clerical workers as one division. In 1961 CIII was not subdivided, but population and mortality data were provided for each Occupation Order, and CIIIc was estimated by use of data for Order XXI: Clerical Workers, 93% of whom formed a strict CIIIc equivalent (O.P.C.S., 1966). For continuity the same procedure was followed in 1971.

Each Decennial Supplement provides numbers of stomach cancer deaths and populations by class in the 10-year age groups 25–34, 35–44, 45–54 and 55–64. Deaths under age 25 are too few for analysis, and over the age of 64 class-specific population and mortality data (where available) tend to be unreliable. Male death rates in these 4 age groups were calculated for the 6 classes, with sub-division of CIII around 1931, 1951, 1961 and 1971, and for the 5 classes around 1921 with CIIIc included in CII. In 1931 deaths were recorded by age for each social class, but in the tables covering occupation groups (including clerical workers) data were given only for cancers of the stomach and oesophagus combined. Stomach-cancer deaths and death rates for clerical workers were estimated by assuming that in each age-group their death rate for oesophageal cancer was the same as for the whole of CIII, and by subtracting this rate from their combined stomach–oesophagus cancer rate.

Single women with occupations are too few to study in this way, but since 1931 the Decennial Supplements have supplied similar mortality data on married women classified by their husbands' social class. Divorced and widowed women are excluded, the population data being restricted to women currently married at census dates, and the mortality data to women currently married at the time of death. Age- and class-specific rates were calculated for married women following the same procedure as for men. In 1931 the published data did not distinguish the wives of clerical workers, and the population of these women was estimated by assuming that in each age-group the ratio of clerical workers to wives was the same as for all men and married women in CIII; stomach-cancer death rates (and numbers of deaths) for clerical workers' wives were estimated by assuming that in 1930–32 their age-specific death rates bore the same ratio to those of all CIII wives as in 1949–53.

The separate stomach-cancer death rates for each sex and class over 1921 to 1971 were examined, but trends tend to be obscured by unstable rates at younger ages due to small numbers of deaths, and by anomalies in occupation classifications which blurred the differences between CI and CII, and CIV and CV. The classes were therefore combined into 2 groups: the first (termed "non-manual"), comprising CI, CII and CIIIc, and the second (termed "manual"), comprising CIIIo, CIV and CV; these terms are broadly accurate, though some non-manual workers such as sales staff, policemen and draughtsmen are included in CIIIo.

### RESULTS

Fig. 1 presents death rates by sex in 10-year age groups for 10-year calendar periods; rates are plotted on a logarithmic scale to permit visual comparison of relative differences by age, date and sex. Although a decline in male rates started around 1931 at all ages under 75, this was generally very slight until at least a decade later. The subsequent decline has been steepest at ages 25–34, where from 1926–35 to 1976–79 the rate has dropped by 78%; the relative decline has been smaller at each subsequent age-group, and at ages 75–84 the rate has fallen by only 13%. Among women an unequivocal decline in rates commenced from 1931 except at ages 25–34, and over the age of 45 the decline has been steeper than among men;

FIG. 1.—Stomach-cancer death rates per 100,000 by sex and age, England and Wales, 1916–1980. ●——● men; ●---● women.

FIG. 2.—Stomach-cancer death rates per 100,000 for grouped social classes at census dates. ●——● men, manual classes; ○——○ men, non-manual classes; ●----● married women, manual classes; ○----○ married women, non-manual classes; □ rate based on fewer than 20 deaths.

in the 2 oldest age groups women's rates converge with those of men 10 years younger. Clearly the sex ratios of age-specific death rates have not been constant, and the pattern noted by Griffith (1968) was characteristic of a particular period; *i.e.* 1958–63. Since then sex ratios have increased at ages over 55, whilst earlier in 1916–25 a very different pattern prevailed, with a ratio of about 1·5 in each age group from 25 to 64. By 1931 the ratio had narrowed to 1·2 at ages 25–34, but had increased to 1·6 or 1·7 between ages 35 and 64.

Fig. 2 presents death rates at census dates for men and married women in manual classes (IIIo, IV, V) and non-manual classes (I, II, IIIc). The vertical scale is interrupted and repeated to avoid overlap of rates at adjacent age groups. These rates and the relevant populations and deaths are set out in Appendix Tables A and B. As indicated, a few of the rates in the age group 25–34 are based on small numbers and are unstable, especially those for non-manual classes in 1971.

Fig. 2 shows that the decline in death rates became slower with increasing age in each sex/class group; it also shows that men in the manual (lower) classes had consistently higher death rates than men in the non-manual (upper) classes in each age-group at each date, and that the same was true of married women. Since 1951, differences between the rates for manual and non-manual classes have generally been larger for men than for married women, but this was not so in 1931. Rates for male manual workers fell relatively slightly from 1931 to 1951, whilst rates for men in non-manual work and for both groups of married women generally fell sharply. There is some overlap of male and female death rates at ages 25–34 (and at ages 35–44 in 1971) with the wives of manual workers having higher rates than men in non-manual occupations.

### DISCUSSION

Improvements in diagnosis have probably confused stomach-cancer mortality trends up to the 1950s, with the disease having been under-certified particularly among the elderly (Doll, 1956; Tulinius, 1979). This view is supported by an apparent drop in rates over age 70 during the 1939–45 war and a subsequent rise, which show most clearly in quinary quinquennial rates (Institute of Cancer Research, 1976); Stocks (1953) regarded the drop as an artefact due to wartime conditions. This study, however, is concerned mainly with ages under 65 and with relative differences in rates; whilst one cannot dismiss any possible effect of improved diagnosis, the divergent trends found for manual workers and their wives make it unlikely that class differences in diagnostic efficiency can account for the main features shown.

Caution is needed for different reasons when interpreting comparisons of social-class rates or ratios at different dates, for although the basic class definitions have remained unaltered, there have been changes at every census in the occupations allocated to different classes, and shifts of large groups of workers from one class to another may affect rates or ratios (R.G., 1958, 1971). Anomalies have also occurred at certain dates; in 1951, for example, CI mortality was inflated by the inclusion of numerous deaths of "company directors" who were mostly enumerated in CII in the census (R.G., 1971). However, the grouping of the classes into the 2 categories of manual and non-manual occupations overcomes most of such shifts and anomalies, and the approximations involved in calculating rates for the small class of clerical workers are unlikely to have distorted rates for the 2 large categories. The grouping also largely overcomes problems of small numbers, and might have uses in studying class trends in mortality from other diseases.

The findings on female class-specific mortality are of necessity limited to married women, whose stomach-cancer death rates at ages 25–64 are lower than those of widows (R.G., 1961; O.P.C.S., 1971), though somewhat higher than those

TABLE.—*SMRs for stomach cancer by social class*, 1921–1971

| Men Class I | II | III | IV | V | Date | (Ages) | Married women Class I | II | III | IV | V |
|---|---|---|---|---|---|---|---|---|---|---|---|
| 60 | 82 | 100 | 106 | 130 | 1921 | (20–64) | — | — | — | — | — |
| 55 | 83 | 98 | 112 | 122 | 1931 | (35–64) | 49 | 77 | 105 | 106 | 121 |
| 57 | 70 | 101 | 112 | 130 | 1951 | (20–64) | 68 | 80 | 102 | 110 | 119 |
| 49 | 63 | 101 | 114 | 163 | 1961 | (15–64) | 50 | 76 | 103 | 110 | 153 |
| 50 | 66 | 109* | 125 | 147 | 1971 | (15–64) | 60 | 84 | 111* | 123 | 145 |

* 1971 SMRs for class III non-manual and manual workers were respectively 79 and 118 for men, and 76 and 122 for married women.

of single women (R.G., 1938, 1958, 1971). However, the understatement of female rates in Fig. 2 due to the omission of widows is trivial in relation to male/female differences and unlikely to have affected trends: in 1965–67 married women's rates were about 3% lower than those of All Women at ages 35–44, 5% lower at 45–54, and 6% lower at 55–64 (O.P.C.S., 1971).

As remarked, the Registrar General's social-class SMRs shown in the Table are heavily weighted by deaths in the age group 55–64, but Fig. 2 shows that in fact class gradients and trends are similar in each age-group. However, Fig. 2 reveals an interesting feature of stomach-cancer mortality trends, which could not be deduced from SMRs alone: that among men in the manual classes rates fell only slightly from 1931 to 1951, in contrast to marked falls for men in the non-manual classes and both groups of married women; only since 1951 have male manual workers' rates fallen at about the same speed as those of the other 3 groups, and at ages 55–64 their rate of decline is still slower. The changing pattern of the ratios of male/female rates in Fig. 1 has been noted, but that figure gives no indication that in the non-manual classes these sex ratios are smaller and have increased less since 1931 than those for manual workers and their wives. Because the manual classes predominate in the population it is principally their experience which is reflected in Fig. 1; Fig. 2 reveals that at ages 25–34 social class is a more important determinant of mortality than sex.

How should one interpret the finding that the stomach-cancer death rates of male manual workers behaved differently during 1931 to 1951 from those of their wives and those of non-manual workers? Why should the decline in rates have gained momentum later among men in the manual classes? There is no obvious answer to this question, for we are not certain why stomach-cancer death rates started to decline around 1931, though most writers who have studied trends favour a change in dietary patterns as the most likely explanation (Doll, 1956; Haenszel & Correa, 1975; Tulinius, 1979). Allowing for the latent period typical of environmental cancers this general hypothesis suggests that some beneficial dietary changes became effective at roughly the time of the 1914–18 war. This is not implausible, for the malnutrition which had earlier been rife among the poorer classes was by then being reduced by various measures, including the introduction of free school meals for many children. During the war there was full employment and many civilian workers had meals provided at works canteens; food rationing and control probably conferred positive dietary benefits on the working classes (Burnett, 1966). During the 1920s all classes must have benefited when various foods became more readily and cheaply available, and there was a rise in the average consumption of fruit and vegetables; items which appear to be negatively associated with stomach cancer (Tulinius, 1979). The more rapid fall in death rates among the young might be due to their shorter unfavourable experience before the beneficial changes took effect, whereas the elderly would have accumulated many decades of adverse experience. Clearly the beneficial changes are continuing ones, for the progressive drop in death rates has not slackened among those born subsequently. It is interesting to note that death rates among white men and women in the U.S.A. differed little from those in this country as late as 1930, and started to decline at roughly the same time, but the U.S.A. rates fell more rapidly (Haenszel, 1958), and by 1958–63 were less than half those in England and Wales.

The delayed decline in rates among manual workers must be linked either to their dietary habits or to some other factor, or to a combination of both. If diet alone was responsible it would be puzzling that the rates for manual workers' wives did not behave similarly: one would have to conjecture that the wives gained relatively more benefit than their husbands from dietary changes. Alternatively (or in addition) it is possible that a proportion of manual workers' stomach cancers are of occupational origin, and that a reduction in such cases occurred relatively late. It has been suggested that men in occupations involving high levels of exposure to dust and fumes generally have above-average stomach-cancer rates (O.P.C.S., 1978), and the possible role of occupation is one of many questions that remain unanswered about the aetiology of gastric cancer. Another such question is why class differentials in rates failed to diminish in either sex from 1951 to 1971, despite the levelling effect on the classes of the 1939–45 war and subsequent social changes.

The figures were prepared by Miss Jean Miller. The Institute of Cancer Research receives support from the Medical Research Council and the Cancer Research Campaign.

### REFERENCES

BURNETT, J. (1966) *Plenty And Want.* London: Thomas Nelson & Sons. p. 214–257.

DOLL, R. (1956) Environmental factors in the aetiology of cancer of the stomach. *Gastroenterologia*, **86**, 320.

GRIFFITH, G. W. (1968) The sex ratio in gastric cancer and hypothetical considerations relative to its aetiology. *Br. J. Cancer*, **22**, 163.

HAENSZEL, W. (1958) Variation in incidence of and mortality from stomach cancer, with particular reference to the United States. *J. Natl Cancer Inst.*, **21**, 213.

HAENSZEL, W. & CORREA, P. (1975) Developments in the epidemiology of stomach cancer over the past decade. *Cancer Res.*, **35**, 3452.

INSTITUTE OF CANCER RESEARCH (1976) *Serial Mortality Tables: Neoplastic diseases Vol.* 1, *England and Wales*, 1911–1970. London: Institute of Cancer Research.

OFFICE OF POPULATION CENSUSES AND SURVEYS (1966) *Census* 1961. *Occupation Tables*, *Table* 3. London: H.M.S.O.

O.P.C.S. (1971) *The Registrar General's Statistical Review of England and Wales for the Year* 1967. *Part III*, *Commentary.* London: H.M.S.O.

O.P.C.S. (1975) *Census* 1971, *Great Britain: Economic Activity Part II, and additional unpublished table DT353U.* London: H.M.S.O.

O.P.C.S. (1978*a*) *Occupational Mortality* 1970–72, *England and Wales.* London: H.M.S.O.

O.P.C.S. (1978*b*, 1979, 1980, 1980) *Mortality Statistics, Cause*, 1976, 1977, 1978, 1979. London: H.M.S.O.

REGISTRAR GENERAL (1927) *Decennial Supplement: England and Wales*, 1921. *Part II: Occupational Mortality, Fertility, and Infant Mortality.* London: H.M.S.O.

R.G. (1938) *Decennial Supplement: England and Wales*, 1931. *Part IIa: Occupational Mortality.* London: H.M.S.O.

R.G. (1958) *Decennial Supplement: England and Wales*, 1951. *Occupational Mortality, Part II.* London: H.M.S.O.

R.G. (1961) *Statistical Review of England and Wales for the Year* 1959. *Part III, Commentary.* London: H.M.S.O. p. 164.

R.G. (1971) *Decennial Supplement: England and Wales*, 1961. *Occupational Mortality Tables.* London: H.M.S.O.

STOCKS, P. (1953) A study of the age curve for cancer of the stomach in connection with a theory of the cancer producing mechanism. *Br. J. Cancer*, **7**, 407.

TULINIUS, H. (1979) Epidemiology of gastric cancer. *Nutr. Cancer*, **1**, 61.

APPENDIX TABLE A

*Populations, stomach-cancer deaths and death rates per* 100,000 *for broad social-class groups around census dates: Men*

| Age group | Class group | | Date 1921–23 | 1930–32 | 1949–53 | 1959–63 | 1970–72 |
|---|---|---|---|---|---|---|---|
| 25–34 | Non-manual | Popln | 518,510 | 555,051 | 643,864 | 708,020 | 906,650 |
| | | Dths | 25 | 34 | 36 | 21 | 6 |
| | | Rate | 1·61 | 2·04 | 1·12 | 0·59 | 0·22* |
| | Manual | Popln | 1,995,590 | 2,474,125 | 2,447,209 | 2,077,970 | 1,966,890 |
| | | Dths | 138 | 161 | 214 | 132 | 44 |
| | | Rate | 2·31 | 2·17 | 1·75 | 1·27 | 0·75 |
| 35–44 | Non-manual | Popln | 575,907 | 584,985 | 779,703 | 853,300 | 888,140 |
| | | Dths | 138 | 128 | 227 | 163 | 69 |
| | | Rate | 7·99 | 7·29 | 5·82 | 3·82 | 2·59 |
| | Manual | Popln | 1,848,853 | 1,904,718 | 2,482,967 | 2,185,270 | 1,864,170 |
| | | Dths | 649 | 725 | 1,255 | 795 | 289 |
| | | Rate | 11·70 | 12·69 | 10·11 | 7·28 | 5·17 |
| 45–54 | Non-manual | Popln | 528,024 | 569,519 | 708,136 | 902,520 | 938,380 |
| | | Dths | 490 | 559 | 846 | 699 | 359 |
| | | Rate | 30·93 | 32·72 | 23·89 | 15·49 | 12·75 |
| | Manual | Popln | 1,559,602 | 1,708,302 | 2,138,114 | 2,228,860 | 1,914,750 |
| | | Dths | 1,982 | 2,352 | 4,304 | 3,546 | 1,255 |
| | | Rate | 42·36 | 45·89 | 40·26 | 31·82 | 21·85 |
| 55–64 | Non-manual | Popln | 354,081 | 438,705 | 520,412 | 695,510 | 792,830 |
| | | Dths | 963 | 1,182 | 1,915 | 1,939 | 1,120 |
| | | Rate | 90·66 | 89·81 | 73·60 | 55·76 | 47·09 |
| | Manual | Popln | 983,602 | 1,297,570 | 1,467,516 | 1,758,500 | 1,784,620 |
| | | Dths | 3,480 | 4,616 | 8,138 | 8,635 | 4,458 |
| | | Rate | 117·93 | 118·58 | 110·91 | 98·21 | 83·27 |

* Based on < 20 deaths.

APPENDIX TABLE B

*Populations, stomach-cancer deaths and death rates per* 100,000 *for broad social-class groups around census dates: Married women*

| Age group | Class group | | Date 1930–32 | 1949–53 | 1959–63 | 1970–72 |
|---|---|---|---|---|---|---|
| 25–34 | Non-manual | Popln | 439,100 | 572,394 | 643,840 | 764,550 |
| | | Dths | 19 | 30 | 17 | 12 |
| | | Rate | 1·44* | 1·05 | 0·53* | 0·53* |
| | Manual | Popln | 1,759,920 | 1,983,459 | 1,764,860 | 1,603,560 |
| | | Dths | 110 | 138 | 107 | 32 |
| | | Rate | 2·08 | 1·39 | 1·21 | 0·67 |
| 35–44 | Non-manual | Popln | 544,254 | 696,926 | 803,210 | 803,800 |
| | | Dths | 99 | 146 | 127 | 49 |
| | | Rate | 6·06 | 4·19 | 3·16 | 2·03 |
| | Manual | Popln | 1,668,686 | 2,081,835 | 1,920,970 | 1,546,370 |
| | | Dths | 441 | 567 | 364 | 135 |
| | | Rate | 8·81 | 5·45 | 3·79 | 2·91 |
| 45–54 | Non-manual | Popln | 498,619 | 615,889 | 772,450 | 818,950 |
| | | Dths | 264 | 383 | 342 | 147 |
| | | Rate | 17·65 | 12·44 | 8·85 | 5·98 |
| | Manual | Popln | 1,382,511 | 1,735,709 | 1,851,500 | 1,562,420 |
| | | Dths | 1,102 | 1,503 | 1,180 | 421 |
| | | Rate | 26·57 | 17·32 | 12·75 | 8·98 |
| 55–64 | Non-manual | Popln | 318,404 | 404,873 | 545,510 | 605,050 |
| | | Dths | 457 | 680 | 606 | 353 |
| | | Rate | 47·84 | 33·59 | 22·22 | 19·45 |
| | Manual | Popln | 872,196 | 1,133,763 | 1,309,610 | 1,292,710 |
| | | Dths | 1,810 | 2,671 | 2,430 | 1,177 |
| | | Rate | 69·17 | 47·12 | 37·11 | 30·35 |

* Based on < 20 deaths.